# Structural Mechanisms of Voltage Sensing in G Protein-Coupled Receptors

**DOI:** 10.1016/j.str.2016.04.007

**Published:** 2016-06-07

**Authors:** Owen N. Vickery, Jan-Philipp Machtens, Giulia Tamburrino, Daniel Seeliger, Ulrich Zachariae

**Affiliations:** 1 Computational Biology, School of Life Sciences, University of Dundee, Dow Street, Dundee DD1 5EH, UK; 2 Physics, School of Science and Engineering, University of Dundee, Nethergate, Dundee DD1 4NH, UK; 3 Forschungszentrum Jülich GmbH, Institute of Complex Systems, Zelluläre Biophysik (ICS-4), Leo-Brandt-Strasse, 52428 Jülich, Germany; 4 Lead Identification and Optimization Support, Boehringer Ingelheim Pharma GmbH & Co KG, 88397 Biberach an der Riss, Germany

## Abstract

G-protein-coupled receptors (GPCRs) form the largest superfamily of membrane proteins and one-third of all drug targets in humans. A number of recent studies have reported evidence for substantial voltage regulation of GPCRs. However, the structural basis of GPCR voltage sensing has remained enigmatic. Here, we present atomistic simulations on the δ-opioid and M2 muscarinic receptors, which suggest a structural and mechanistic explanation for the observed voltage-induced functional effects. The simulations reveal that the position of an internal Na^+^ ion, recently detected to bind to a highly conserved aqueous pocket in receptor crystal structures, strongly responds to voltage changes. The movements give rise to gating charges in excellent agreement with previous experimental recordings. Furthermore, free energy calculations show that these rearrangements of Na^+^ can be induced by physiological membrane voltages. Due to its role in receptor function and signal bias, the repositioning of Na^+^ has important general implications for signal transduction in GPCRs.

## Highlights

• We investigate high-resolution GPCR structures under voltage in MD simulations
• We find evidence for charge movement in agreement with experimental gating charges
• The voltage-sensing mechanism involves a cation moving in a hydrated pocket
• Migration of the ion is predicted to have major functional effects on GPCRs

## Graphical Abstract

**Figure:**
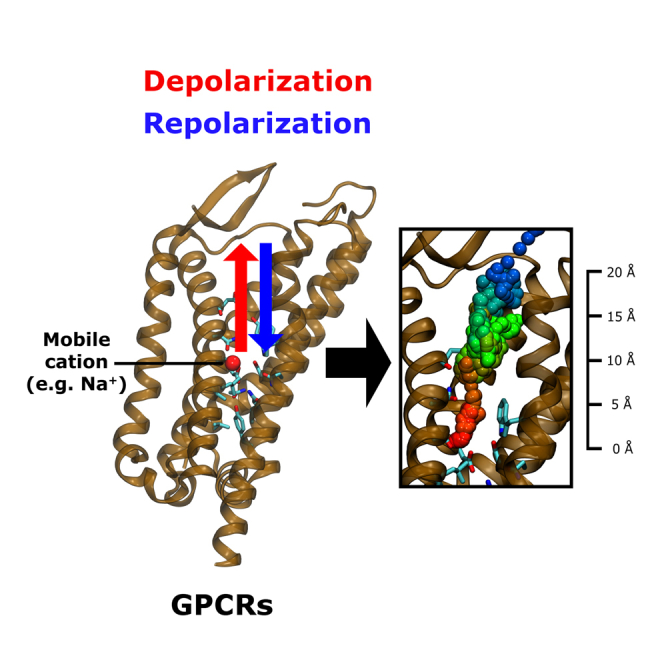

## Teaser

Vickery et al. describe a new voltage-sensing mechanism, which is suggested to underpin electric regulation of class A GPCRs. Molecular dynamics simulations characterize the voltage-driven movement and energetics of an internally bound cation, which gives rise to gating charges in excellent agreement with experiments.

## Introduction

Membrane voltage (V_m_) is an intrinsic property of all cell membranes, with a physiological range between about −100 and +150 mV (Kandel et al., 2000). Whereas all cells have a resting V_m_, excitable cells such as neurons undergo rapid changes between negative and positive V_m_ during neurotransmission (Reyes, 2001). In addition, differences in resting V_m_ have been reported for a variety of cell types and different phases in the cell cycle (Yang and Brackenbury, 2013). Despite an extensive body of work on voltage-gated ion channels, the effect of V_m_ on the function and conformational changes of other membrane proteins has not been as widely investigated. G-protein-coupled receptors (GPCRs) form the largest group of integral membrane proteins in the human genome (Lagerström and Schiöth, 2008), facilitating downstream propagation of extracellular binding information into intracellular signal transduction cascades (Pierce et al., 2002). Due to this function, GPCRs are the therapeutic targets of more than one-third of all available drugs (Hopkins and Groom, 2002).

Recently it has been shown that physiologically relevant V_m_ can elicit functional or conformational effects in several independent GPCRs (Martinez-Pinna et al., 2004, Rinne et al., 2013, Ben Chaim et al., 2013; for review, see Mahaut-Smith et al., 2008). Furthermore, electrophysiological recordings of the α_2A_ adrenergic and M1 and M2 muscarinic receptors (M1R and M2R) have revealed a voltage-induced rearrangement of charges (gating currents) when the receptors were exposed to depolarized membrane voltages (Ben-Chaim et al., 2006, Navarro-Polanco et al., 2011, Rinne et al., 2013). In particular, evidence for the movement of a gating charge of ∼0.50–0.85e has been obtained for the M1R and M2R (Ben-Chaim et al., 2006, Navarro-Polanco et al., 2011). Most of the measurements made on wild-type (WT) and mutant M2Rs converge to a gating charge near 0.5e (Navarro-Polanco et al., 2011). Recent work by Rinne et al. (2015) has shown that V_m_ modulates the receptor signal transduced into the cell in a way that depends on the nature and binding pose of agonists within the orthosteric pocket, and therefore an interaction of the voltage sensor with the orthosteric ligand binding pocket has been inferred. Despite all of these observations, however, the nature of the GPCR voltage sensor has so far remained elusive (Rinne et al., 2015).

Here, we address the structural and functional consequences of V_m_ on two GPCRs by atomistic molecular dynamics (MD) simulations under voltage. Due to its clinical importance and exclusive distribution in excitable CNS tissues, where it is frequently exposed to large changes in V_m_, we first investigate the δ-opioid receptor (δ-OR). In addition, we look at the muscarinic receptor M2R, on which most of the previous measurements of charge rearrangements have been performed (Ben-Chaim et al., 2006, Navarro-Polanco et al., 2011).

**Figure 1:**
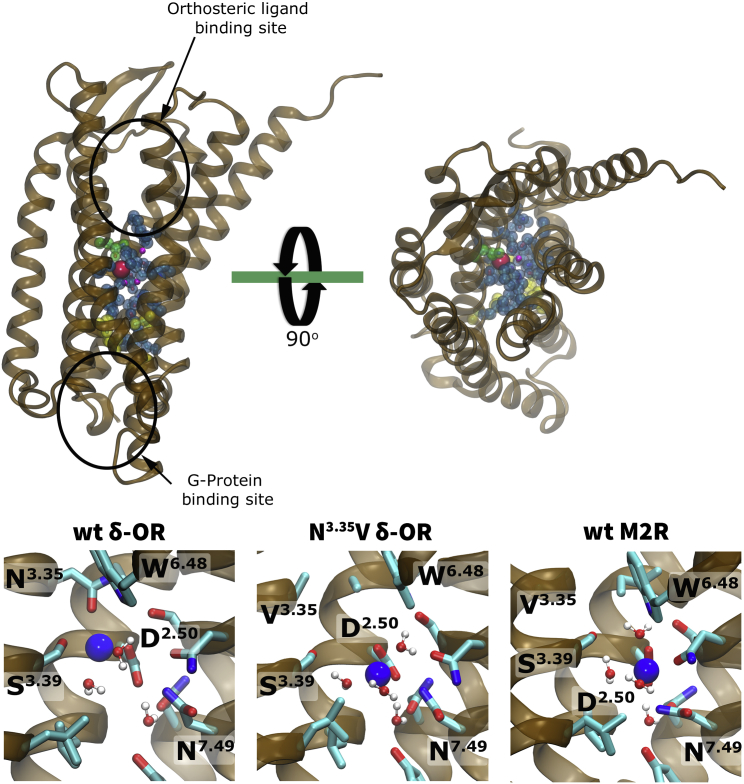
Conserved Residues Forming the Internal Hydrophilic Pocket of Class A GPCRs (Top) Sixteen conserved residues (blue/red spheres) line the hydrophilic pocket in GPCRs (Katritch et al., 2014). The pocket connects the orthosteric ligand binding site and the G-protein binding site (black ellipses). The pocket is accessible from the extracellular side but separated from the intracellular side by a hydrophobic layer (Yuan et al., 2014) (yellow spheres). Fifteen of the 16 residues are highly conserved (blue); the less conserved position 3.35 is shown in green. At the allosteric binding site for sodium, water molecules are depicted as purple spheres (water oxygen atoms), and the Na^+^ ion is shown in red. The receptor is shown in side view on the left; the right panel displays a top view from the extracellular side. (Bottom) Close-up views of the binding site for Na^+^ (blue sphere) within the hydrophilic pocket of the WT δ-OR, Asn131^3.35^Val δ-OR, and the WT M2R, as observed in our simulations. The binding site for Na^+^ in the M2R was inferred from the position and interactions of the ion in the WT δ-OR and the conservation level of the Na^+^ binding residues.

The δ-OR crystal structure (PDB: 4N6H) reveals a Na^+^ ion situated within the central core of the receptor transmembrane (TM) domain (Fenalti et al., 2014). It is bound near the base of a water-filled hydrophilic pocket, which extends from the orthosteric ligand binding site to the conserved and functionally important NP^7.50^xxY motif near the G-protein binding site (Figure 1) (Pardo et al., 2007). The binding site for Na^+^, formed by the residues Asp95^2.50^, Asn131^3.35^, and Ser135^3.39^ (superscript numbers refer to the Ballesteros and Weinstein residue numbering system [Ballesteros and Weinstein, 1995]) in the δ-OR, is highly conserved across all rhodopsin-like GPCRs, including the muscarinic receptors (Katritch et al., 2014). It is therefore thought that Na^+^ binding to this site occurs in all or most class A GPCRs (Katritch et al., 2014, Massink et al., 2015), although it is not normally unequivocally detected in crystal structures of lower resolution, for example the M2R (PDB: 3UON; Haga et al., 2012). Among the 16 pocket-lining residues, only sequence position 3.35 is less conserved, showing a nearly equal partition between hydrophilic and hydrophobic residues (Asn in δ-OR, Val in M2R, Ala in M1R; see Figure 1) (Vroling et al., 2010). Previous MD simulations and experimental mutagenesis work on the adenosine A_2A_ receptor have revealed the importance of the highly conserved hydrophilic pocket residues in Na^+^ binding and receptor function (Massink et al., 2015). Na^+^ has long been known to allosterically modulate a wide range of GPCRs, with large effects on ligand binding, agonist efficacy, and signal bias (for review, see Katritch et al., 2014). In previous MD simulations, Na^+^ binding to Asp^2.50^ drastically reduced the flexibility of the receptors, possibly confining them to inactive conformations, while the absence of Na^+^ increased receptor dynamics and sampling of the active state (Miao et al., 2015, Selent et al., 2010). These findings indicate a key functional role for the Na^+^ ion and its position within class A GPCRs.

Our results show that the internal Na^+^ ion in GPCRs is the most movable charge in the receptors under voltage. The position of the ion in the hydrophilic pocket strongly depends on V_m_, and the voltage-related repositioning of the Na^+^ ion generates a gating charge in excellent agreement with the experimentally obtained values. The nature of the pocket-lining residues can influence its motion, and it is possible that other cations could replace the Na^+^ ion. Furthermore, we show that large-scale conformational changes of the receptor alone give rise to much smaller gating charges, far below the experimental observations. Our data therefore suggest that the movement of a charge in the internal water-filled pocket of GPCRs along the membrane normal is the most plausible mechanism to explain the voltage-related effects observed in GPCRs.

## Results

### Computational Electrophysiology Simulations Reveal Voltage-Induced Mobility of Allosteric Na^+^

We first conducted a series of atomistic MD simulations based on a double bilayer setup (Sachs et al., 2004) and the computational electrophysiology (CompEL) protocol (Kutzner et al., 2011, Kutzner et al., 2016) on WT and mutant δ-OR at depolarized V_m_, with a total simulated time of >30 μs. We applied electrochemical Na^+^ gradients across the membrane to evoke V_m_ between ∼250 and ∼1,000 mV to accelerate any effects on the limited simulation timescale. Due to its conservation as an ionizable residue and close interaction with a cation in the crystal structure, the side chain of Asp95^2.50^ at the main binding site for Na^+^ was modeled in a negatively charged protonation state. Furthermore, we found that protonation of the side chain of Asp95^2.50^ in test simulations reduced the affinity of the allosteric site for Na^+^ such that the ion spontaneously dissociated from the receptor on very short timescales, which is incompatible with the crystallographically observed binding of Na^+^ within the hydrophilic pocket.

**Figure 2:**
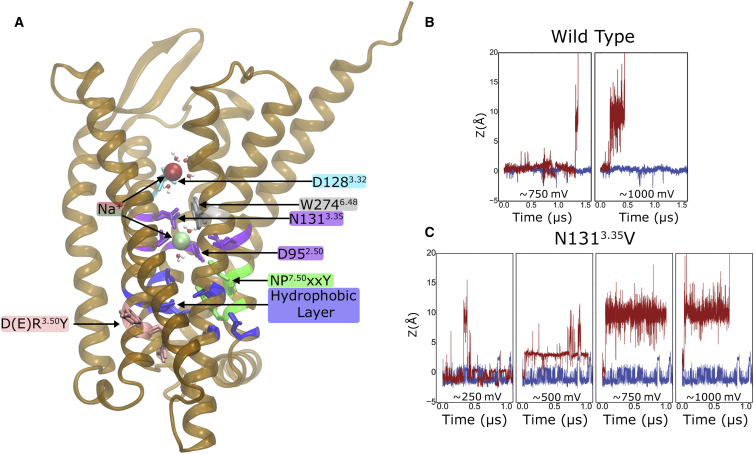
Effect of Depolarized V_m_ on the Position of the Na^+^ Ion in δ-OR (A) Structure of the δ-OR depicting Na^+^ bound in the allosteric (green) and orthosteric binding sites (red). The binding sites correspond to coordinates of z = 0 Å and z = ∼9 Å in the graphs shown in (B) and (C), respectively. (B and C) Z coordinate of the internal Na^+^ ion in WT δ-OR (B) and in the Asn131^3.35^Val mutant (C) under depolarized V_m_, displaying translocation of the Na^+^ ion induced by V_m_ (depolarized V_m_ simulations, red trace; 0 mV control, blue trace). The approximate V_m_ at the time of the transitions is noted in the graphs. Key functionally important residues are shown as sticks and color-coded into the groups: D(E)R^3.50^Y motif (pink), hydrophobic layer (blue), NP^7.50^xxY motif (green), allosteric Na^+^ binding pocket (magenta), “toggle switch” residue Trp274^6.48^ (silver), Na^+^ binding site in the orthosteric pocket (cyan).

Our simulations reveal that the location of the Na^+^ ion in δ-OR, and thereby its coordination pattern with the protein, is strongly influenced by the electric field (Figure 2). Figure 2B shows the position of the internal Na^+^ ion along the TM axis (z) in WT δ-OR in response to depolarized V_m_. When the V_m_ exceeds ∼700 mV, the Na^+^ ion is expelled from its allosteric binding site in the inner core of the TM domain, and moves to the orthosteric ligand binding site of WT δ-OR. At this site, the Na^+^ ion is coordinated by the side chains of Asn131^3.35^, Asp128^3.32^, and the backbone of Asp128^3.32^. A secondary binding position is located somewhat further toward the extracellular side, where the ion mainly interacts with the side-chain oxygen atoms of Asp128^3.32^. From the orthosteric binding position, the ion can leave the receptor completely and enter into the extracellular aqueous solution (Figure 2B). Under negative or neutral V_m_, no movement of the Na^+^ ion toward the extracellular side is recorded on equivalent or longer timescales (see Figures S1 and S2).

In experiments, the δ-OR Asn131^3.35^Val mutation decreases the allosteric effect of Na^+^ in δ-OR and lowers the Na^+^ dependence of the δ-OR agonists, while retaining a mild binding affinity for Na^+^ (Fenalti et al., 2014). We therefore mutated position 3.35 to Val to study the effect of a pocket-lining hydrophobic residue on the voltage-induced movement of the Na^+^ ion. The mutation also accounts for the fact that sequence position 3.35 is variable between hydrophilic and hydrophobic amino acids among class A GPCRs, whereas the remaining 15 pocket-lining residues are highly conserved (Katritch et al., 2014).

**Figure 3:**
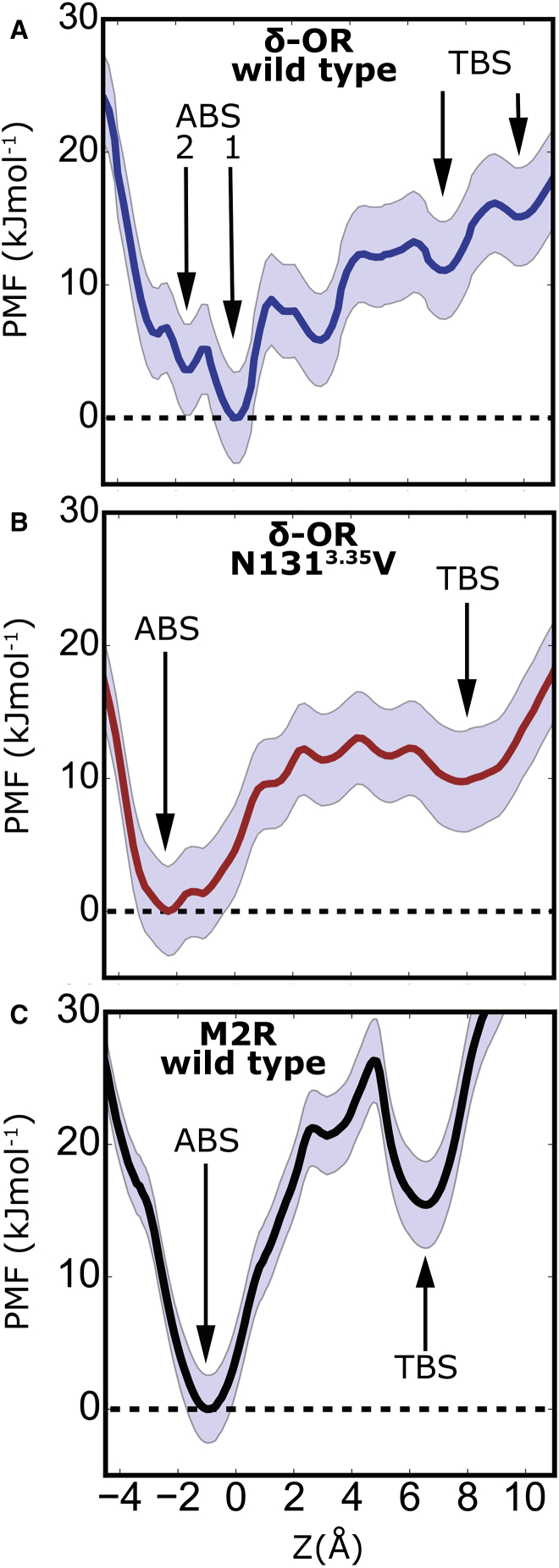
Potential-of-Mean-Force Profiles of Na^+^ Translocation in the Hydrophilic Pocket of WT δ-OR, Asn131^3.35^Val δ-OR, and WT M2R (A–C) Equilibrium potential of mean force (PMF) of Na^+^ translocation along the TM axis z in WT δ-OR (A), the Asn131^3.35^Val mutant (B), and the M2R (C). Arrows highlight key binding sites; the SD obtained from bootstrap analysis is depicted as light-blue shading. The observed energy barriers for movement of a Na^+^ ion within the hydrophilic pocket are low, and in the range of the energy provided by physiological and experimental membrane voltages. ABS, allosteric binding site; TBS, transient binding site.

The simulations of the Asn131^3.35^Val δ-OR mutant reveal a much larger susceptibility of Na^+^ to the influence of V_m_ (Figure 2C). In contrast to the WT, V_m_ above ∼250 mV is now sufficient to transiently displace Na^+^ from the allosteric binding pocket in the mutant. Membrane voltages above 500 mV remove Na^+^ from the inner core of the TM domain of the mutant within a time span of <0.1 μs. As observed for the WT, voltage-induced dissociation of Na^+^ from the internal pocket usually leads to reassociation with Asp128^3.32^ in the orthosteric ligand binding site. Our simulations also show that the mutation modifies the nature of the allosteric Na^+^ binding site, reflected by fluctuations between the crystallographic binding site (z = 0 Å) and a location about 2 Å deeper within the internal binding pocket (Figure 2C). In both the WT and mutant simulations, the exit trajectory for Na^+^ through the orthosteric ligand binding site follows a pathway similar to that of previous simulations, in which random accelerated MD were used to enforce the exit of Na^+^ without the application of V_m_ (Shang et al., 2014). In our simulations, the dual internal Na^+^ binding sites remain occupied for the duration of the simulation under the absence of V_m_ (Figure S1). Upon application of a more negative (hyperpolarized) V_m_, the Na^+^ ion is transiently attracted deeper into the hydrophilic pocket to a second binding site in WT-δ-OR, while within the Asn131^3.35^Val mutant the Na^+^ ion becomes stabilized in a deeper binding position (Figure S2). Interestingly, at large negative V_m_ a second cation is observed to enter the hydrophilic pocket in the WT, occupying both the major and minor energy minima (as defined by the potential of mean force [PMF] shown in Figure 3A) over substantial time spans. Figure S3 shows the dual occupation of the pocket by Na^+^ ions at highly negative potentials.

### Energetics of Ion Movement within the Internal Pocket

As our initial simulations revealed that application of supra-physiological levels of depolarized V_m_ consistently resulted in Na^+^ migration from the internal allosteric binding pocket to the orthosteric ligand binding site on the simulation timescale, we next investigated whether V_m_ on experimental and physiological levels is sufficient to drive this movement. We quantified the equilibrium free energy barrier for the transition by recording the PMF of Na^+^ along the TM axis *z*, using umbrella sampling in the absence of V_m_.

In WT δ-OR, the PMF reveals a total free energy barrier of 13.0 ± 2.5 kJ mol^−1^ for this transition (Figure 3A). The major free energy minimum is identical to that of the Na^+^ binding site defined in the δ-OR crystal structure (Fenalti et al., 2014), with Asn131^3.35^, Ser135^3.39^, and Asp95^2.50^ as main coordinating residues (z = 0 Å). A second free energy minimum at the base of the pocket is situated somewhat further toward the intracellular side, near z = −1.6 Å, where the ion is coordinated by the side chains of Asn310^7.45^, Asn314^7.49^, Asp95^2.50^, and the Leu91^2.46^ backbone (Figure 3A). The two major minima are separated by an energy barrier of 5.3 ± 2.5 kJ mol^−1^. The presence of the orthosteric Na^+^ binding site near Asp128^3.32^ is reflected in local energy minima near z = ∼7 Å and z = 10 Å (here termed the transient binding site). The main energy barrier for movement between the allosteric and transient binding sites arises from the constricted passage of the ion between the side chains of Asn131^3.35^ and Trp274^6.48^. Furthermore, a local energy barrier of 9.0 ± 2.5 kJ mol^−1^ confines the ion to its crystallographic binding site in extracellular direction. This barrier corresponds to a movement of the Asn131^3.35^ side chain toward the orthosteric ligand binding pocket, which is required for efficient transfer of the Na^+^ ion.

The PMF of the Asn131^3.35^Val mutant displays a similar free energy barrier for Na^+^ movement from the internal allosteric binding pocket to the orthosteric binding site of 13.0 ± 2.5 kJ mol^−1^ (Figure 3B). However, the energy barrier is wider and exhibits a less rugged shape than in WT δ-OR, reflecting the formation of a more hydrophobic gate that separates the orthosteric and allosteric pockets in the mutant. The allosteric Na^+^ binding site is also broadened, thus generating a binding region of ∼4 Å diameter, and the most preferred binding position is located deeper within the allosteric Na^+^ binding pocket (z = −2.2 Å; Figure 3B). Overall, the mutation facilitates the movement of Na^+^ within the inner pocket, which also leads to an increased fluctuation level of the ion under the absence of V_m_ (Figure S1B).

Our analysis shows that binding of Na^+^ to the transient binding site in the orthosteric pocket is only ∼11 kJ mol^−1^ higher in energy than binding to the allosteric position in the case of WT δ-OR, and ∼10 kJ mol^−1^ in the case of the Asn131^3.35^Val mutant. Moreover, movement of the Na^+^ ion within a range of ∼6 Å in the δ-OR allosteric pocket (both WT and mutant) experiences energy barriers below 10 kJ mol^−1^, while the complete removal of Na^+^ from the allosteric binding site requires the surmounting of only a small additional activation barrier in the region of ∼3 kJ mol^−1^.

This demonstrates that physiologically relevant V_m_ provides sufficient energy to shift the position of the internal Na^+^ ion over substantial distances within the pocket, and to move it between the allosteric and orthosteric binding sites. By comparison, the potential energy of a monovalent ion in a voltage gradient of ∼100 mV amounts to ∼10 kJ mol^−1^.

**Figure 4:**
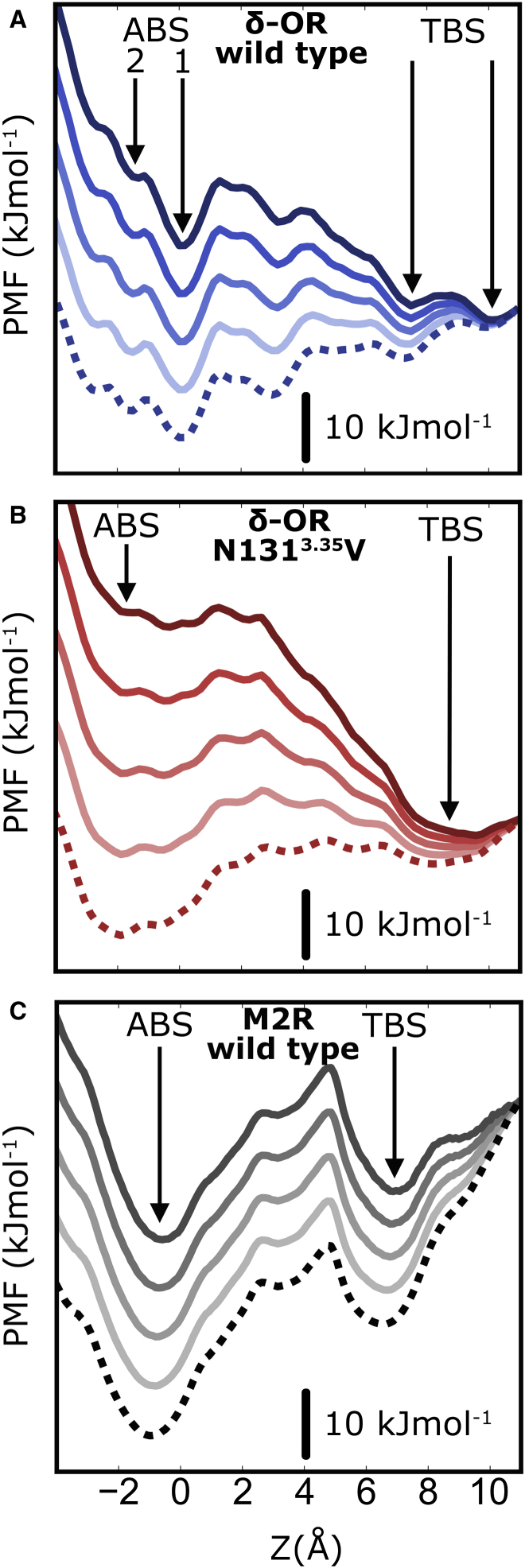
Non-equilibrium Effect of V_m_ on the PMF Profiles of Na^+^ Translocation within the Internal Pocket (A–C) Voltage-induced tilt of the free energy surface of Na^+^ in the non-equilibrium case in WT δ-OR (A), the Asn131^3.35^Val mutant (B), and the M2R (C). Increments are from 250 mV (light) to 1,000 mV (dark); dotted line indicates 0 mV. The underlying voltage drop was mapped by using the gating charge calculations displayed in Figure 6. The removal of kinetic traps on the pathway of the ion in the Asn131^3.35^Val mutant becomes evident from the smoother surfaces in (B). Note that the graphs display relative energy differences for each voltage regime rather than absolute energy values. The black bar therefore denotes an energy difference of 10 kJ mol^−1^ within each curve, and the offset between the curves has been arbitrarily selected. ABS, allosteric binding site; TBS, transient binding site.

The Asn131^3.35^Val mutation generally reduces the ruggedness of the free energy landscape in the pocket and leads to gentler slopes, kinetically facilitating the movement of the Na^+^ ion (Figure 3B). Similar to the effect reported for ion permeation through the CRAC channel (Dong et al., 2013), the smoother energy landscape for Na^+^ ion movement in the Asn131^3.35^Val mutant δ-OR is linked to an increased hydration of the hydrophilic pocket in the mutant from 13 to 18 waters (Figure S4), which also raises the hydration level of the migrating Na^+^ ion. In the non-equilibrium case of applying V_m_, the ruggedness of the energy landscape has important consequences for the rate of transitions (Figure 2C). Both the steeper slopes of the energy barriers and the increased roughness of the energy surface contribute to the formation of kinetic traps upon tilting of the energy surface, which arises from the voltage drop across the membrane (Hyeon and Thirumalai, 2003, Nevo et al., 2005). Figure 4 displays the effect of the voltage drop experienced by the Na^+^ ion along the axis of the internal pocket at various V_m_ levels. The detailed shape of the voltage gradient inside the pocket was determined from gating charge calculations of ion movement along the axis (see the section Determination of Gating Charges below). Depolarization results in a major change of the energy surface in the pocket, with the orthosteric site becoming the global energy minimum for Na^+^ binding in the receptor cavity in all studied receptors. Our findings also show that the presence of Val^3.35^ in the δ-OR mutant accelerates outward transition of Na^+^ by exhibiting fewer kinetic traps, although the main equilibrium energy barrier is similar to that of WT δ-OR.

It is important to note that throughout all simulations and receptor types, we continuously see binding and dissociation events of Na^+^ ions at the transient binding site on the simulation timescales, regardless of V_m_. However, this movement occurs on a multitude of different pathways, precluding the single-collective coordinate representation of the PMF, which is an appropriate description of the transition pathway only within the inner pocket.

To compare the energy landscapes with another receptor type, we next studied the M2R. While no experimental data are presently available for the δ-OR, evidence for the movement of gating charges within M2R has previously been obtained through voltage-clamp recordings.

Compared with the δ-OR we followed a slightly different protocol, as the lower resolution of the M2R crystal structure (3.0 Å) (Haga et al., 2012) precluded the detection of an Na^+^ ion at the conserved ion binding site (Asp69^2.50^) in M2R. However, Na^+^ has been inferred to be present in M2R as well, based on the conservation level of the binding site and on functional considerations (Katritch et al., 2014). Also, the number of water molecules detected within the inner pocket is much smaller in the crystal structure of M2R (4) compared with the crystal structure of the δ-OR (16). As has been previously shown, however, Na^+^ ions from the external solution are found to bind spontaneously to Asp^2.50^ in MD simulations (Miao et al., 2015, Selent et al., 2010), and additional water molecules enter the pocket on a timescale of nanoseconds. We therefore initially simulated the M2R until the pocket was completely hydrated, and attracted an Na^+^ ion into the allosteric binding site by using a hyperpolarized V_m_. We then calculated the PMF of the observed transition pathway. As Figure S5 shows, the inward transition pathway for the ion is highly similar to the outward pathway, taken by the ion from the allosteric pocket to the transient binding site, as determined from our PMF calculations.

The PMF of Na^+^ in the hydrophilic M2R pocket displays a clearer distinction between the two ion binding sites (Figure 3C). The free energy barrier separating binding at the allosteric from the transient orthosteric binding site, here at residue Ile72^2.53^, is slightly higher at ∼26 ± 2.5 kJ mol^−1^. We attribute the larger local free energy barrier to a tighter hydrophobic constriction of the Na^+^ transition pathway within the orthosteric ligand binding pocket by Ile72^2.53^ approximately 4.5 Å above the Asp69^2.50^ side chain. The minor energy well present at z ∼ 3 Å is due to the coordination of the allosteric Na^+^ with an asparagine and two serine side chains (S107^3.36^, S433^7.46^, and N432^7.45^).

The energy difference between the allosteric and transient binding sites is comparable with that of the WT δ-OR (∼15 kJ mol^−1^). This means that a similar duality between two ion binding sites of similar free energy exists for the M2R and δ-OR in the case of depolarized V_m_ (see Figure 4C). The higher energy barrier between the two sites in the M2R would give rise to a slightly higher activation energy under V_m_ for this transition; however, the expected rates for overcoming a barrier of this size would still be rapid on physiologically relevant timescales.

It is worth noting that the lower resolution of the M2R X-ray structure with respect to the δ-OR structure, and the fact that some receptor sections were not modeled in the original M2R structure, might incur a slightly higher level of inaccuracy in the M2R PMF. Generally, however, the PMF of Na^+^ in the M2R shows a remarkable similarity to the free energy profile in the δ-OR.

### Determination of Gating Charges

To further characterize the repositioning of Na^+^ under the influence of V_m_, we investigated the gating charge that arises from the movement of the ion between the floor of the hydrophilic pocket, and the base and the top of the orthosteric binding site in the WT δ-OR and the M2R (Figure 6). We used a novel protocol to calculate gating charges in proteins, which has recently been developed and optimized for use in conjunction with CompEL double membrane setups (J.P.M., R. Briones, B. de Groot, Ch. Fahlke, unpublished data). A similar method optimized for ionic imbalances across single bilayers has previously been successfully employed to calculate gating charges in K^+^ channels (Treptow et al., 2009, Delemotte et al., 2011).

In brief, the TM voltage obtained for a single insulated or double bilayer system under electrochemical gradients is a function of the charge imbalance in the bulk solutions on either side of the membrane (Δq_bulk_) and charge imbalances within the membrane-immersed protein (Δq_prot_) (Delemotte et al., 2011). At a given bulk charge imbalance, differences in V_m_, averaged over time, therefore originate from a rearrangement of the charge distribution within the protein, corresponding to a measurable gating charge (Delemotte et al., 2011) (see also Figure S6).

Figure 6A shows that the movement of the Na^+^ ion from the allosteric to the top of the orthosteric binding site (and further into the extracellular bulk solution) gives rise to a maximum gating charge of 0.42 ± 0.03e, 0.63 ± 0.03e, and 0.53 ± 0.02e for the WT δ-OR, Asn131^3.35^Val δ-OR, and WT M2R, respectively. For movement to regions near the base of the orthosteric pocket, our calculations predict gating charges of up to ∼0.3e for the WT δ-OR and WT M2R. Experimentally determined gating charges on M2R span a range from ∼0.5 to 0.85e (Navarro-Polanco et al., 2011, Ben-Chaim et al., 2006). However, the majority of experimental values obtained on WT and mutant M2R cluster around values of ∼0.5e. For the α_2A_ adrenergic receptor, a gating charge of about 0.5e has been reported. The gating charges predicted from our computations are thus in excellent agreement with the majority of gating charges measured previously in M2R and other GPCRs (Ben-Chaim et al., 2006, Navarro-Polanco et al., 2011, Rinne et al., 2013).

Our results also show that the movement of a charge or ion inside the GPCR structure must cover a substantial distance in the direction of the membrane normal to result in a gating charge near 0.5e (see Figure 6). More spatially restricted rearrangements of a charged group are unlikely to explain such a large value for the gating charge. Notably, even under a V_m_ of 1,000 mV, we have not observed the extensive movement of any other charged residue in the receptors.

**Figure 5:**
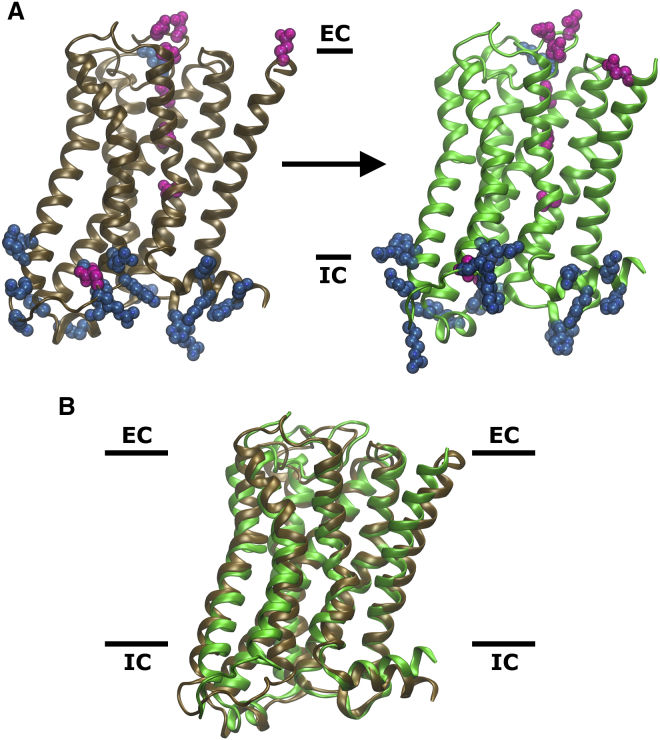
Maximal Movement of Charged Protein Residues upon the Transition of M2R from the Inactive to the Activated Conformation (A) Conformational change of the M2R from the inactive state (left, brown) to the active state (right, green), representing an upper limit to the known receptor conformational dynamics and the movement of charged residues. Bars denote the membrane limits (EC, extracellular; IC, intracellular side). Charged residues are shown as spheres for clarity (positive, blue; negative, magenta). A small gating charge of only ∼0.13e is linked to the activation transition of the receptor. (B) Comparison of the overall conformation of inactive (brown) and active M2R (green; PDB: 3UON and 4MQT, respectively).

To substantiate this notion, we tested the maximum gating charge that would be related to the receptor change of conformation from the inactive to the active form, which can be assumed to reflect an upper boundary to the conformational variability of the protein. The M2R has been crystallized in both inactive and active conformation (Haga et al., 2012, Kruse et al., 2013). Using the same protocol described above, the transition from the inactive to the activated state of M2R leads to a gating charge of 0.13 ± 0.02e if Na^+^ movement is disregarded. This transition includes the entire protein and therefore entails conformational changes of the charged residues at the DR^3.50^Y motif, Glu^6.30^, and Asp^3.32^ (Figure 5). The calculated value is therefore too small to serve as an explanation for the experimentally observed charge movements in GPCRs. Our data show that they are more likely to arise from a more extended charge movement along the axis of the internal hydrophilic pocket, which spans a large portion of the bilayer thickness.

**Figure 6:**
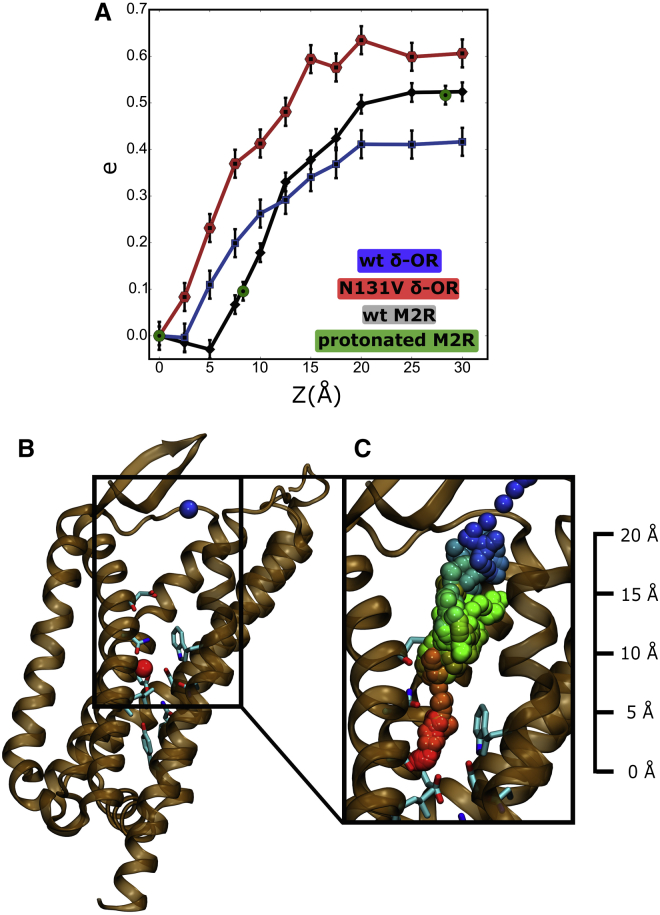
Gating Charge Resulting from the Movement of a Na^+^ Ion or a Proton from the Allosteric Pocket to the Bulk Solution via the Orthosteric Binding Site (A) Gating charge arising from the movement of Na^+^ within the internal pocket of the M2R (black), WT δ-OR (blue), and the Asn131^3.35^Val δ-OR (red), calculated with a step size of 2.5 Å. The maximal gating charges are 0.42e, 0.53e, and 0.63e for the WT δ-OR, WT M2R, and Asn131^3.35^Val δ-OR, respectively. The green circles show the gating charge that would arise from a similar transfer of a proton from Asp^2.50^ to the extracellular surface of the M2R via Asp^3.32^ and Asp173 (∼0.5e). (B) Na^+^ positions corresponding to the allosteric binding site and the top of the orthosteric binding pocket (the Na^+^ is colored red and blue respectively). (C) Pathway taken by the Na^+^ ion from the hydrophilic pocket to the extracellular bulk solution in the δ-OR (color-coded according to simulation time proceeding from red to blue); TM helix 5 has been omitted for clarity. The errors were estimated from the variation within the slopes of the charge-voltage relationships (see Figure S6). See also Figure S7.

Other charges, however, can be envisaged to undergo the same movement, which would lead to an experimentally indistinguishable gating current. We therefore additionally tested the hypothesis that protonation changes within the hydrophilic pocket can give rise to the recorded gating charges. The pK_A_ of ionizable groups residing in the TM domains of a protein depends on V_m_ (Kralj et al., 2011), and changes in V_m_ could therefore alter the protonation state of these residues. The allosteric Na^+^ site is formed by the highly conserved residue Asp^2.50^ located approximately in the center of the TM section. Its protonation state has been addressed in a number of previous studies (Zhang et al., 2013, Zhang et al., 2014, Isom and Dohlman, 2015), and its pK_A_ has been calculated to be close to neutral pH (Ranganathan et al., 2014). Unless a cation such as Na^+^ binds to Asp^2.50^, this side chain could therefore become protonated, and exhibit a protonation state sensitive to V_m_. The orthosteric binding site includes a further highly conserved ionizable side chain, Asp^3.32^. We calculated the gating charge for the transfer of a proton from Asp^2.50^ via Asp^3.32^ to the boundary between the orthosteric binding pocket and the external solution in the M2R (by deprotonation of Asp^2.50^ and protonation of Asp^3.32^ and Asp173^ECL2^, respectively; Figure 6A, green circles). As can be seen, a voltage-induced proton transfer from Asp^2.50^ across the internal pocket of M2R to the extracellular space would result in the recording of a gating charge identical to that of the movement of an Na^+^ ion along the same distance.

## Discussion

While the physiological importance of V_m_ in all cell types is appreciated (Yang and Brackenbury, 2013, Pardo and Stühmer, 2014), the effect of V_m_ on the structure and function of most integral membrane proteins remains sparsely investigated. In recent years, increasing evidence supporting a direct influence of V_m_ on the activity of GPCRs has been obtained. For instance, V_m_ has been demonstrated to influence agonist-mediated activation of α_2A_ adrenergic receptors (Rinne et al., 2013), agonist binding to the M2 muscarinic receptor (Ben Chaim et al., 2013), and downstream signaling of the P_2_Y_1_ purinergic receptor and M2R (Martinez-Pinna et al., 2004, Ben-Chaim et al., 2003). The movement of gating charges has been demonstrated for the α_2A_ adrenergic and the M1 and M2 muscarinic receptors (Ben-Chaim et al., 2006, Navarro-Polanco et al., 2011, Rinne et al., 2013). As most of these receptors are expressed in excitable tissue, their regulation by V_m_ may be a physiologically important control mechanism (Mahaut-Smith et al., 2008). However, the precise mechanism underlying voltage sensitivity of GPCRs, and the effect of V_m_ on their atomic conformation, has remained elusive so far, including the structural equivalent of the charge movement.

Our microsecond-timescale atomistic simulations of the δ-OR show that the allosteric Na^+^ ion seen in high-resolution crystal structures constitutes the most mobile charge in the receptors under voltage, while the protein itself does not exhibit any significant rearrangement of other charged groups, including the D(E)R^3.50^Y motif and residue Asp^2.50^. Most of the 16 pocket-lining residues are highly conserved in rhodopsin-like (class A) GPCRs, with the exception of sequence position 3.35, which can be occupied by polar groups (e.g., Asn in δ-OR) or hydrophobic residues (e.g., Val in M2R). Our results may therefore bear relevance for most other class A GPCRs, which share the same internal pocket structure.

Our computational studies show that depolarized TM electric potential is capable of moving the allosteric Na^+^ ion from its allosteric binding site near Asp^2.50^ to the orthosteric ligand binding pocket, and demonstrate that this motion generates a maximum gating charge of 0.42–0.63e in the δ-OR and M2R. For both the M2 and M1 muscarinic receptors, a gating charge of 0.55–0.85e has been reported in experiments upon membrane depolarization, and a gating charge of 0.5e has been detected for the α_2A_ receptor (Ben-Chaim et al., 2006, Navarro-Polanco et al., 2011, Rinne et al., 2013). Most experimental measurements for WT and mutant M2R display values near 0.5e (Navarro-Polanco et al., 2011, Ben-Chaim et al., 2006). The excellent agreement between the experimental gating charges and those caused by the movement of the Na^+^ ion, which we observe, therefore provides a plausible structural explanation for the experimentally recorded charge rearrangement. Of note, it has been shown that mutation of Asp^2.50^ to Ala in the M2R abolishes the recording of any gating currents in that receptor, even under voltages of 200 mV (Navarro-Polanco et al., 2011). Although the authors noted that this finding might be attributed to a somewhat lower surface expression of the mutant, they concluded that alternatively the mutation of Asp^2.50^ might cause the absence of gating currents, and that Asp^2.50^ could therefore play a key role in the mechanism of voltage sensing (Navarro-Polanco et al., 2011). Our results are in agreement with the observation that gating charge movements are absent from an Asp^2.50^Ala mutant of the M2R and form strong support for the latter explanation, i.e., the crucial role of the Na^+^ binding site for voltage regulation of GPCRs.

**Figure 7:**
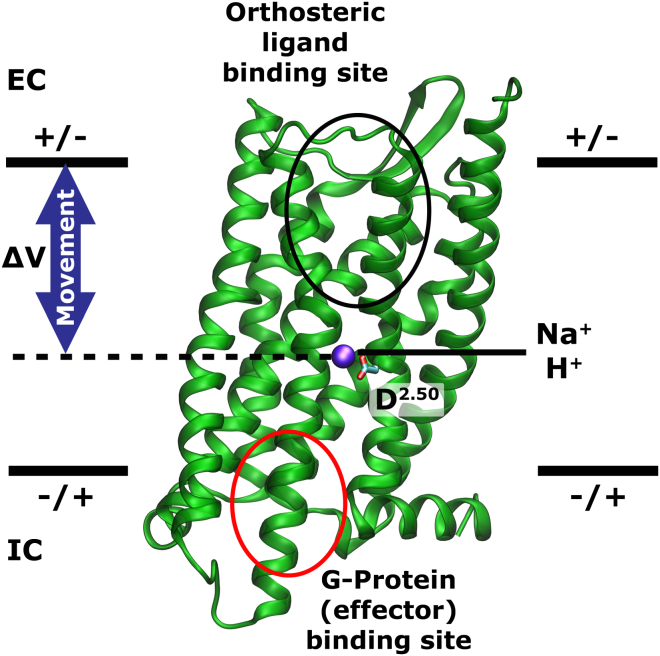
Graphical Representation of the Suggested Voltage-Sensing Mechanism in GPCRs A hydrophilic pocket, open to the extracellular face only, connects the extracellular ligand and the intracellular effector protein binding sites in GPCRs. A Na^+^ ion is bound to a conserved site near the ionizable residue Asp^2.50^. The position of the ion in the pocket strongly responds to changes in membrane voltage, and its movement along the TM axis in the water-filled pocket leads to a gating charge of ∼0.5e when the ion travels from its allosteric binding site to the top of the ligand binding pocket. In Na^+^-free conditions, a nearly identical gating charge can be elicited by a voltage-induced change in the protonation state of Asp^2.50^ and movement of a proton. The side chain of Asp^2.50^ is likely to be in a protonated state if no Na^+^ ion is bound. In both cases, the voltage-induced repositioning of ions involves a change in the ionic interactions with the orthosteric ligand binding site and transmission to the intracellular effector binding site, and therefore functional consequences and ligand interactions are an intrinsic feature of this mechanism.

Furthermore, the movement of the charge has been shown to affect ligand binding in these receptors (Ben-Chaim et al., 2006, Navarro-Polanco et al., 2011, Dekel et al., 2012), which agrees with our observation that Na^+^ shows voltage-induced migration between the allosteric and orthosteric binding sites (Figures 6 and 7). Very recently it has been found that V_m_ modulates the G-protein-dependent and G-protein-independent signal in muscarinic receptors, but that the magnitude and direction of the influence displays a dependence on the precise chemical structure of the agonist and its binding pose in the receptor pocket (Rinne et al., 2015). The voltage-sensing mechanism we propose requires cations to relocate to or traverse the orthosteric pocket, where they would interact with any bound ligand. A strong interplay with the action of agonists is therefore easily conceivable at this site, as well as a dependence of the precise nature of the effect on the molecular detail of the agonists and the pocket under this mechanism.

To investigate further the receptor transition into the active state, we calculated the predicted gating charge for the transition of the M2R from its inactive to its active state conformation, and found values of only ∼0.13e, demonstrating that the movement of a charge over a larger distance normal to the membrane surface is necessary to explain the experimental observations. Notably, our finding that the movement of a cation along the distance of ∼20 Å inside the receptors can explain the majority of experimentally observed gating charges in GPCRs is not restricted to an Na^+^ ion. We have tested the possibility that voltage-induced deprotonation of the conserved acidic residue Asp^2.50^ due to pK_A_ change, and subsequent transfer of a proton toward the extracellular space, could give rise to similar gating currents. As expected, this transition exhibits a gating charge identical to that of an Na^+^ ion over the same distance.

Most of the experimental observations of voltage effects in GPCRs have been made under the presence of Na^+^ in the external solution (Rinne et al., 2013, Navarro-Polanco et al., 2011), while other sets of experiments were conducted at zero external Na^+^ concentration, but in the presence of other cations such as Ca^2+^ and the organic cation N-methyl-D-glucamine (Ben-Chaim et al., 2006). It has recently been shown that the organic cation amiloride is able to bind directly to the sodium binding site at Asp^2.50^ and induce an allosteric effect similar to that of Na^+^ ion (Massink et al., 2015). Furthermore, a possible interaction of other monovalent and divalent cations such as K^+^ and Ca^2+^ with the internal pocket has been indicated in previous experimental and simulation studies (Strasser et al., 2015, Pasternak et al., 1975). Under physiological conditions, we consider Na^+^ to be the most likely source of the gating charge due to its high external concentration, and its clear detection bound in close contact to Asp^2.50^ in recent high-resolution crystal structures (Liu et al., 2012, Fenalti et al., 2014). In the absence of Na^+^, however, our results show that other cationic interactions such as a protonation change of the conserved Asp^2.50^ could result in the observation of a gating change of similar magnitude. In that context, it has previously been shown that Asp^2.50^ is likely to exhibit protonation-deprotonation reactions near pH ∼7 when an ion is not bound (Ranganathan et al., 2014). Alternatively, other cations, even larger organic cations, could undergo the same transition if their concentration is sufficiently high to replace Na^+^ (Massink et al., 2015). We currently cannot exclude a more intricate interplay of protonation changes at Asp^2.50^ and movements of cations within the pocket, however, as during each single simulation the protonation state of Asp^2.50^ (and other residues) was fixed. Dynamic protonation changes dependent on the position of Na^+^, for example, could play a further role in voltage sensing and the observation of gating currents. A graphical representation of the GPCR voltage-sensing mechanism proposed by our work is shown in Figure 7.

As the allosteric Na^+^ binding site inside the hydrophilic internal pocket is conserved across all class A GPCRs, with the exception of visual rhodopsins, our results predict that voltage sensitivity and gating currents may be found in other GPCRs and could be a general feature of these membrane proteins. The receptor types we have investigated in this study are expressed mainly in electrically excitable cells such as neurons. It is therefore tempting to speculate that the voltage regulation of excitable-tissue GPCRs plays a physiological role. In this context, it is interesting to consider the similarity and differences to more canonical voltage-sensing domains (VSDs), for instance those commonly found in voltage-gated K^+^ or Na^+^ channels (Souza et al., 2014). While in these VSDs a highly focused electric field, conserved in the sequence of a wide array of VSDs, acts together with the displacement of a usually greater gating charge in total to ensure an exquisite voltage sensitivity in the low-millivolt range, the voltage-sensing mechanism we propose here seems more highly adapted to report larger-scale changes in V_m_ into the signal transduction pathway. Of note, the voltage-sensing mechanism we suggest for GPCRs lies outside the polypeptide chain and involves an ion moving within a conserved protein pocket, as opposed to a charged TM section of the protein itself.

It has been shown by mutations of the ion binding site that the presence or absence of Na^+^ ions at the allosteric binding site near Asp^2.50^ in the δ-OR modifies the signal bias between β-arrestin and G_αi_ upon activation of δ-OR (Fenalti et al., 2014). Our findings show that V_m_ affects the occupation of the allosteric binding site with Na^+^. They therefore indicate that V_m_, and thus the excitation state of the cells, might have an impact on the bias between different receptor signaling pathways in these pharmacologically important GPCRs. Our results suggest a new range of electrophysiological and receptor-functional experiments to test the influence of V_m_ on GPCR signal bias.

## Experimental Procedures

The δ-OR simulation systems were constructed using the crystal structure of the δ-OR (PDB: 4N6H) (Fenalti et al., 2014), from which the BRIL subunit and the antagonist ligand naltrindole were removed. The simulation system of the M2R was constructed from the crystal structure of the M2R (PDB: 3UON) (Haga et al., 2012), from which T4 lysozyme and the antagonist 3-quinuclidinyl-benzilate were removed. The cleaved intracellular loop 3 (ICL3) in M2R was reconnected by using Modeller (v9.14) (Šali and Blundell, 1993). For both systems, all external water and lipid molecules were removed, while all internal water molecules or ions were retained. The charged N- and C-terminal residues were neutralized using acetyl and methyl moieties, respectively. The systems were simulated with default protonation states including a negatively charged Asp^2.50^. The Asn131^3.35^Val δ-OR receptor mutants were generated from the previously edited δ-OR receptor.

Each receptor structure was inserted into a fully equilibrated and hydrated 1,2-palmitoyl-oleoyl-*sn*-glycero-3-phosphocholine (POPC) lipid bilayer using the GROMACS utility g_membed (Wolf et al., 2010) with an overall system size of (∼92 × 88 × 97 Å^3^). A NaCl concentration of 145 mM was used for the aqueous solution. To equilibrate the systems, we position-restrained all protein heavy atoms with a force constant of 1,000 kJ mol^−1^ nm^−2^ for 5–10 ns. Due to the lower resolution and minimal hydration of the M2R crystal structure, this system was then equilibrated for 100 ns without position restraints to enable full hydration of the hydrophilic pocket. We used the amber99sb_ildn force field for the protein (Lindorff-Larsen et al., 2010), Berger parameters for lipids (Berger et al., 1997), which were adapted for use with the amber99sb force field (Cordomí et al., 2012), and the SPC/E model for water molecules (Berendsen et al., 1987). Water bond angles and distances were constrained by SETTLES (Miyamoto and Kollman, 1992) while all other bonds were constrained using the lincs method (Hess et al., 1997). The temperature and pressure were kept constant throughout the simulations at 310 K and 1 bar, respectively, with the protein, lipids, and water/ions coupled individually to a temperature bath by the v-rescale method using a time constant of 0.2 ps and a semi-isotropic Berendsen barostat (Bussi et al., 2007, Berendsen et al., 1984). The use of the virtual site model for hydrogen atoms (Feenstra et al., 1999) allowed the use of 4-fs time steps during the simulation. All simulations were performed with the GROMACS software package, version 4.6 (Hess et al., 2008).

For the CompEL simulations, the aforementioned systems were duplicated along the z axis to construct a double bilayer system, and ionic imbalances from 1 to 4 Na^+^ ions were used between the aqueous compartments to generate a range of TM potentials from ∼250 to ∼1,000 mV, as previously described (Kutzner et al., 2011). We determined the V_m_ by using the GROMACS utility g_potential in overlapping 2-ns time windows with a 1-ns running average throughout the trajectory.

To calculate the PMF for the Na^+^ ion within the allosteric Na^+^ binding pocket at neutral V_m_, we used umbrella sampling together with the GROMACS utility g_wham (Hub et al., 2010). We used bins of <0.5 Å size along the z axis and simulation times of >150 ns in each bin. To record the PMF of ion movement within the pocket in M2R, we first simulated the equilibrated and hydrated membrane/protein system for another 100 ns under a hyperpolarized V_m_ until a sodium ion located to the allosteric binding pocket. The SD for the PMF profiles was generated by using the Bayesian bootstrap method with 200 runs. The free energy minima of the WT, Asn131^3.35^Val δ-OR, and the M2R for Na^+^ were set to G = 0 kJ mol^−1^. Throughout the text the position of the Na^+^ ion (z coordinate) is reported relative to the Asp^2.50^-Cα atom of the relevant receptor. All structural images were produced using VMD v1.92 (Humphrey et al., 1996).

For our calculation of gating charges, the single bilayer system was duplicated along the z axis, with one bilayer inverted (both extracellular components of the receptors facing each other). The charge imbalance across the compartments was then initially neutralized by adding ions. All protein atoms except hydrogen were position-restrained using spring constants of 1,000 kJ mol^−1^ nm^−2^, while the allosteric Na^+^ ion was position-restrained with a spring constant of 10,000 kJ mol^−1^ nm^−2^ due to its increased mobility. Bulk Na^+^ ions were position-restrained on the z axis using a spring constant of 200 kJ mol^−1^ nm^−2^ to avert ingress of Na^+^ from the bulk solution to the orthosteric ligand binding pocket. The systems were simulated with net charge imbalances between −4 and 4, probing the allosteric position of Na^+^ near the base of the hydrophilic pocket (coordinated by Asp^2.50^ and Ser^3.39^) and the position in the orthosteric binding pocket (coordinated by Asp^3.32^). The slopes of the charge imbalance-voltage relationship indicate near-constant capacitance of the membrane/protein system under these conditions (Figure S6). The gating charges were then inferred from the voltage difference observed for each pair of ion positions at a given charge imbalance. The errors were derived from the maximum and minimum slopes of the charge imbalance-voltage relationship.

For the scan of ion position effects on gating charges, the allosteric Na^+^ was positioned at 2.5-Å intervals from the hydrophilic pocket to the extracellular bulk solution and simulated for 50 ns with the first 5 ns discarded. For the gating charge calculations in the case of M2R, a single sodium ion was placed in a position within the allosteric sodium binding pocket identical to the δ-OR, coordinated by Asp69^2.50^ and Ser110^3.39^. For the calculation of the possible gating charge induced by the conformational shift of M2R from inactive to active, we used the same protocol on the inactive (PDB: 3UON^19^) and active structure (PDB: 4MQT^29^). The system was equilibrated for 30 ns before calculating the predicted gating charge arising from the conformational change. The gating charge calculated for each point along the hydrophilic pocket was taken as a direct measure of the shape of the underlying voltage drop within the pocket. This voltage drop, multiplied by *e*, was added to the equilibrium PMF obtained from umbrella sampling, in order to illustrate the effect of V_m_ on the energetics of the Na^+^ ion in the internal pocket in Figure 4.

## Author Contributions

Conceptualization, U.Z.; Methodology, O.N.V., J.-P.M., and U.Z.; Analysis, O.N.V., J.-P.M., G.T., and U.Z.; Investigation, O.N.V.; Resources, J.-P.M. and D.S.; Writing – Original Draft, O.N.V. and U.Z.; Writing – Review and Editing, O.N.V., J.-P.M., G.T., D.S., and U.Z.; Funding Acquisition, D.S., and U.Z.; Supervision, U.Z.

## Supplemental Information

Document S1. Figures S1–S7

Document S2. Article plus Supplemental Information
