# Supplementary material for: Structural Mechanisms of Voltage Sensing in G Protein-Coupled Receptors
Source: Structure. 2016 Jun 7;24(6):997–1007. doi: 10.1016/j.str.2016.04.007 (PMC4906246; doi:10.1016/j.str.2016.04.007)
Supplement: Document S1. Figures S1–S7 [file mmc1.pdf]

Structure, Volume *24*

Supplemental Information

Structural Mechanisms of Voltage Sensing in G Protein-Coupled Receptors

Owen N. Vickery, Jan-Philipp Machtens, Giulia Tamburrino, Daniel Seeliger, and Ulrich Zachariae

Structural Mechanisms of Voltage Sensing in G-Protein Coupled Receptors

Supplementary Material

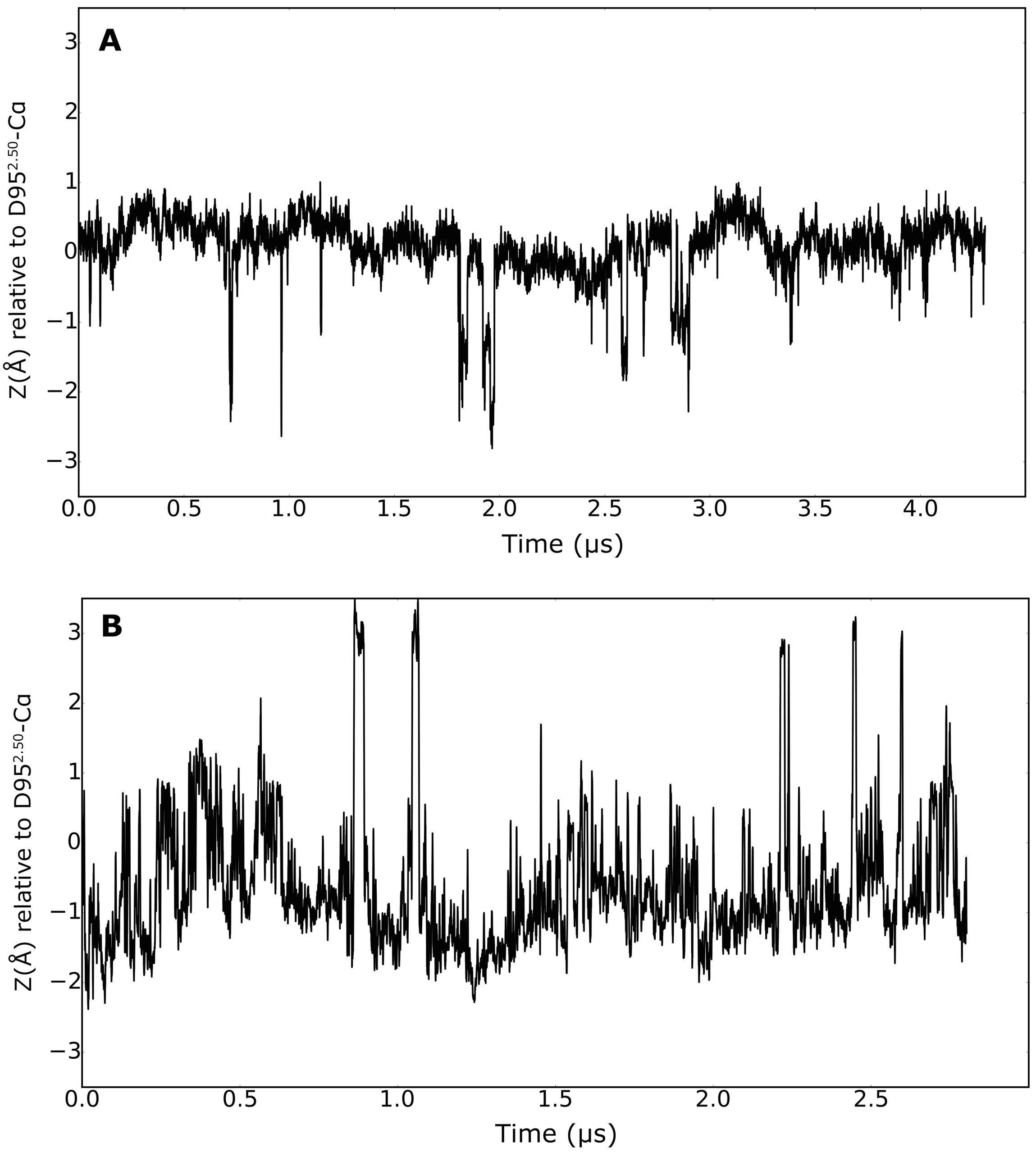

Figure S1 (related to Figure 2): Stable binding of the internal Na$^+$ ion at ABS under a membrane voltage of 0 mV.

Z-coordinate of the allosteric Na$^+$ ion in wt δ-OR (**A**) and the mutant Asn131$^{3.35}$Val δ-OR (**B**) under no V_m as control. The wt δ-OR shows stable coordination of the Na$^+$ ion within the major allosteric Na$^+$ binding site, with minor transient fluctuations into the secondary allosteric Na$^+$ binding site. In the Asn131$^{3.35}$Val δ-OR mutant, the Na$^+$ ion primarily occupies the major energy minima shown in Figure 2B, with some minor fluctuations within the pocket.

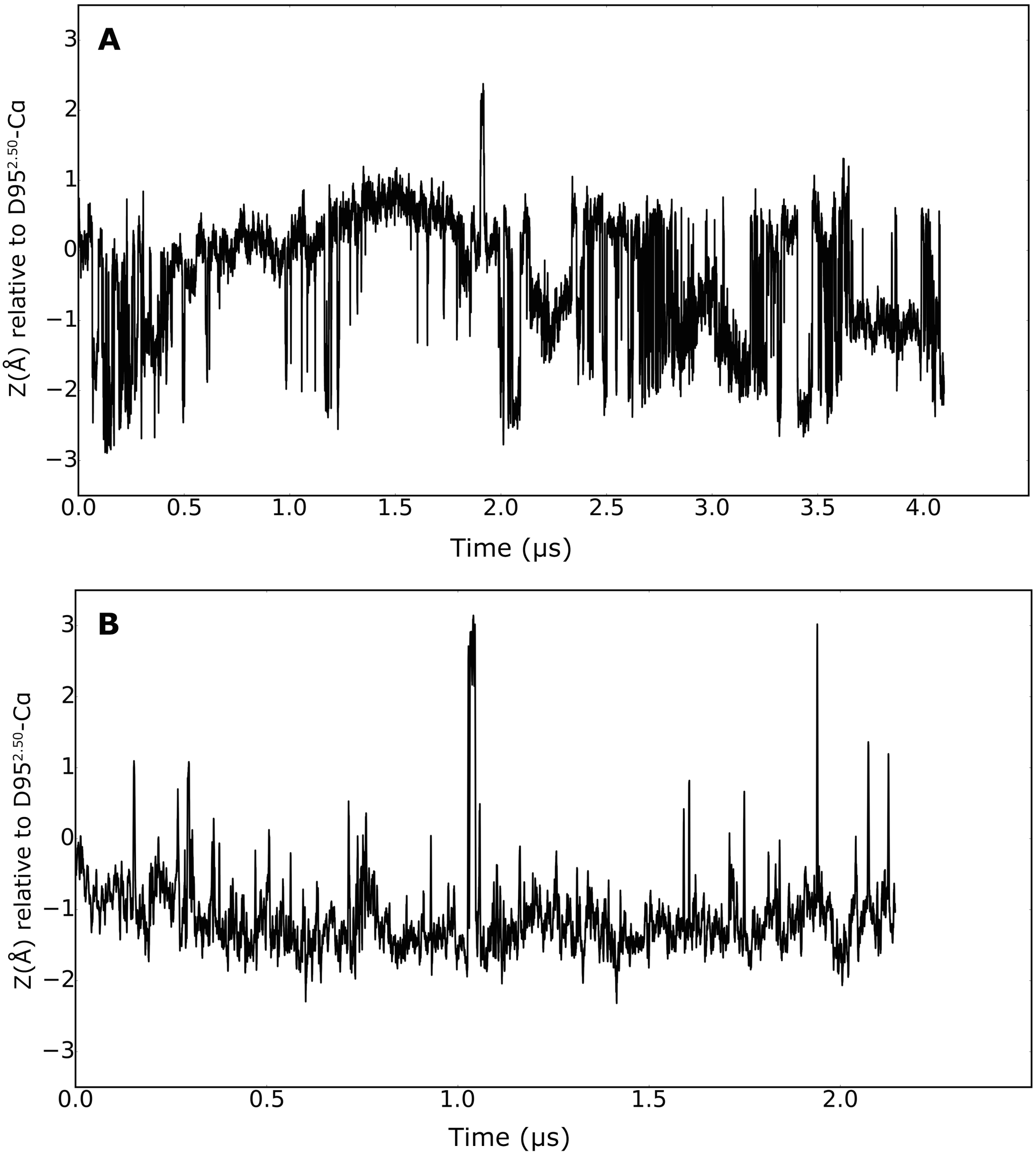

Figure S2 (related to Figure 2): Stable binding of the internal Na^+ ion at ABS under a negative membrane voltage.

Z-coordinate of the allosteric Na^+ ion in wt δ-OR (**A**) and the mutant $Asn131^{3.35}Val$ δ-OR (**B**) under a hyperpolarized V_m. of -500 mV. The allosteric Na^+ ion within the wt δ-OR shows multiple transitions between the major binding site and the secondary allosteric Na^+ binding site. In the $Asn131^{3.35}Val$ δ-OR mutant the Na^+ ion primarily occupies the major energy minima shown in Figure 2B, however is predominantly situated deeper within the hydrophilic pocket with minor upward fluctuations.

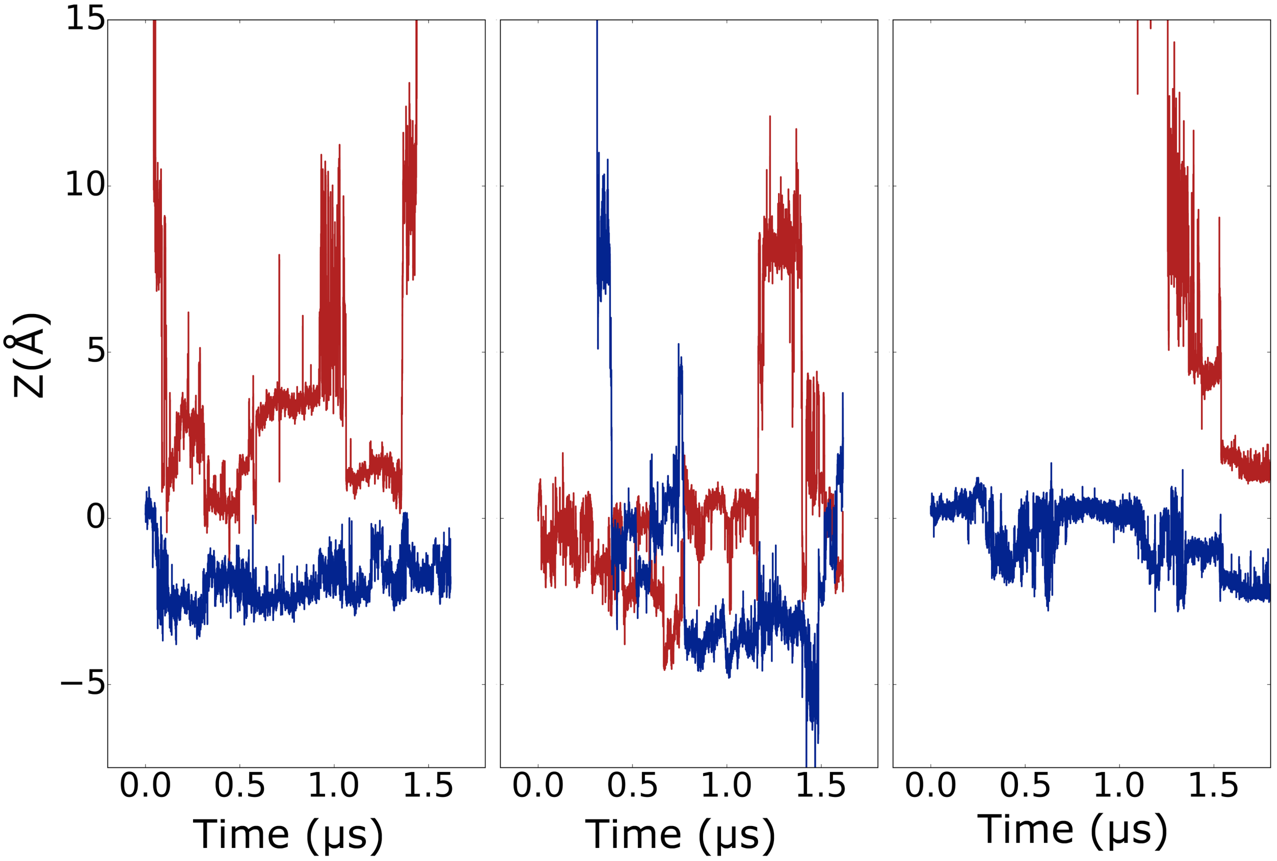

Figure S3 (related to Figure 3): Dual occupancy of the wt δ-OR under negative V_m.

Z-coordinate of internal Na^+ ions within the wt δ-OR under strongly hyperpolarised V_m of 1000mV. The figure shows that the inner hydrophilic pocket can bind two ions simultaneously. The original Na^+ ion predominantly resides within the secondary allosteric Na^+ binding site (blue trace), and is coordinated by the side chains of $Asn310^{7.45}$, $Asn314^{7.49}$ $Asp95^{2.50}$ and the $Leu91^{2.46}$ backbone. This provides enough space for the major binding site to be occupied by a secondary Na^+ ion (red trace), coordinated by the sidechains of $Asn131^{3.35}$, $Ser135^{3.39}$ and $Asp95^{2.50}$. The dual occupation with Na^+ persists for the majority of the simulation time. In these simulations, the second Na^+ ion can either exit the hydrophilic pocket to the extracellular side (Left) or can switch positions with the original allosteric Na^+ ion (Middle).

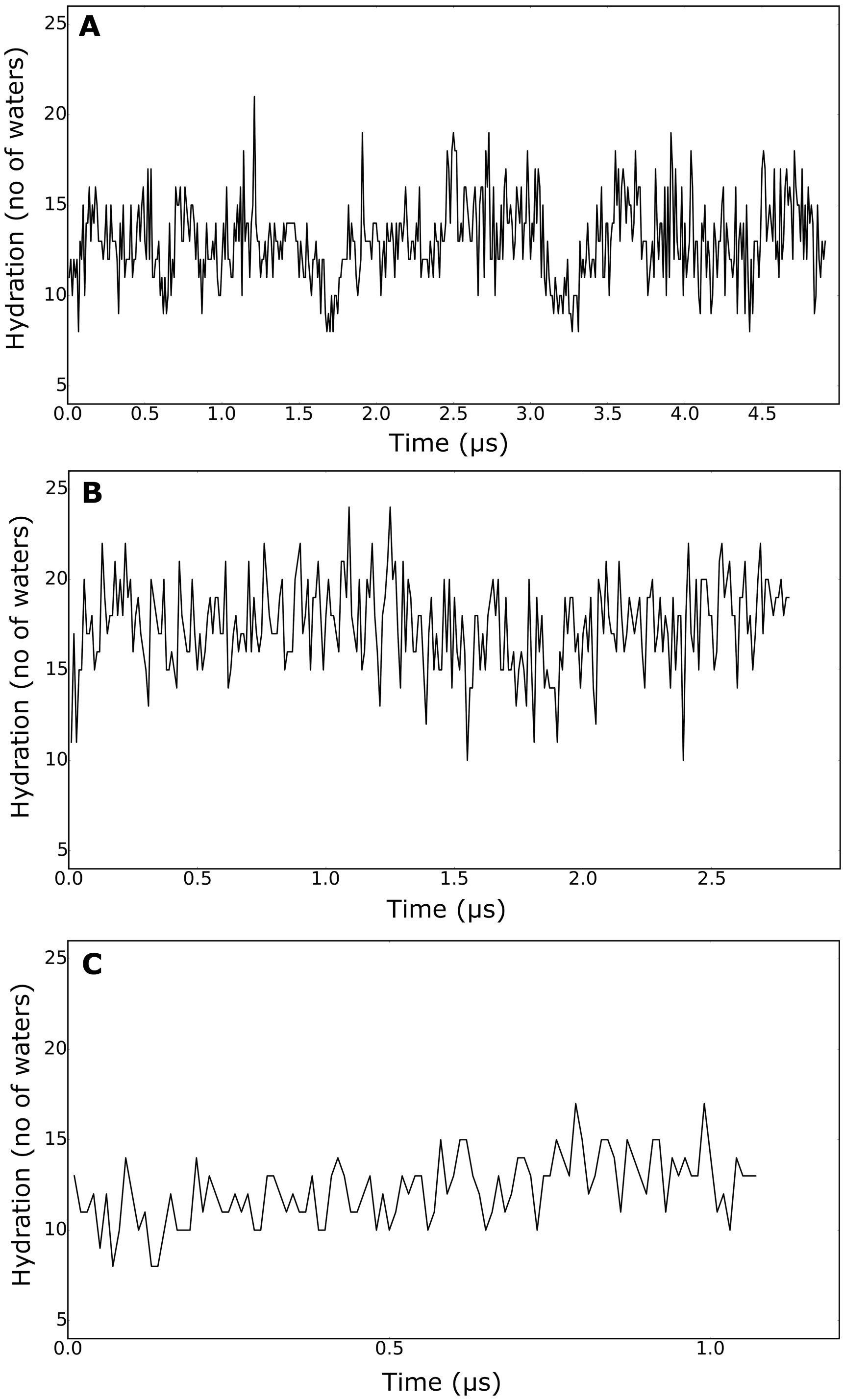

Figure S4 (related to Figure 3): Hydration of the hydrophilic pocket.

The number of water molecules within the hydrophilic pocket shows stable hydration levels over micro-second time-scale simulations. A similar mean hydration level is observed between wt δ-OR (**A**) and m2r (**C**) of 13.0±2.2 and 12.2±1.8 respectively. However the mutation of Asn131$^{3.35}$ to Val leads to an increased hydration level of 17.4±2.4 water molecules in the pocket. The number of water molecules was determined every 10ns from simulations at 0 mV between limits defined by the atoms Y$^{7.53}$-Cα and D$^{3.32}$-Cα (±sd).

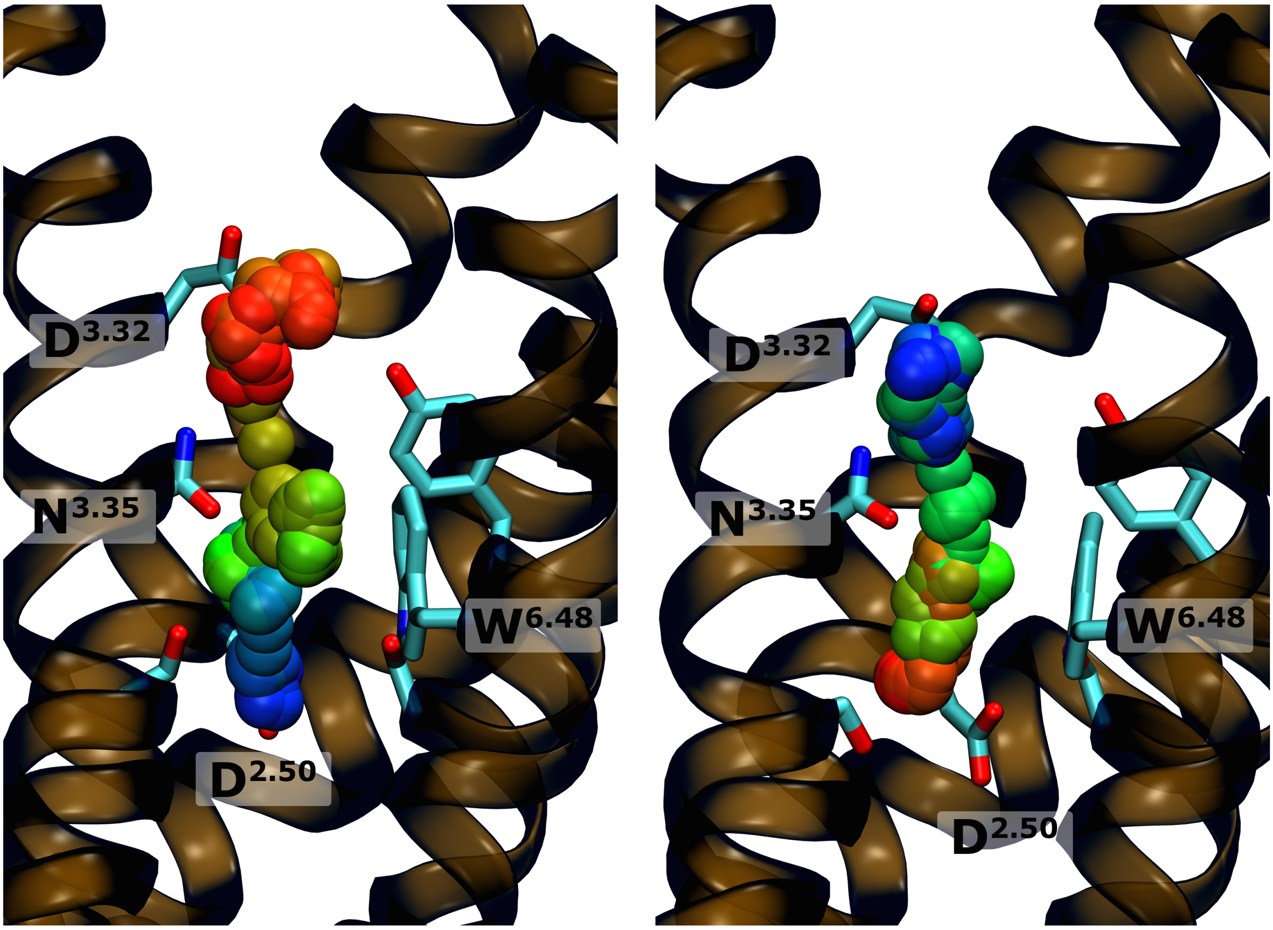

Figure S5 (related to Figure 3): Comparison of inward and outward pathways of Na^+.

Pathways taken by the Na^+ ion from the transient binding site to the hydrophilic pocket (left) or from the hydrophilic pocket to the transient binding site (right) in the wt δ-OR (color-coded according to simulation time proceeding from red to blue). In both directions, the Na^+ ion passes centrally between $N131^{3.35}$ and $W^{6.48}$, where it is coordinated by the oxygen atom from the N131 sidechain and a cation-π interaction between the ion and the aromatic sidechain of $W^{6.48}$.

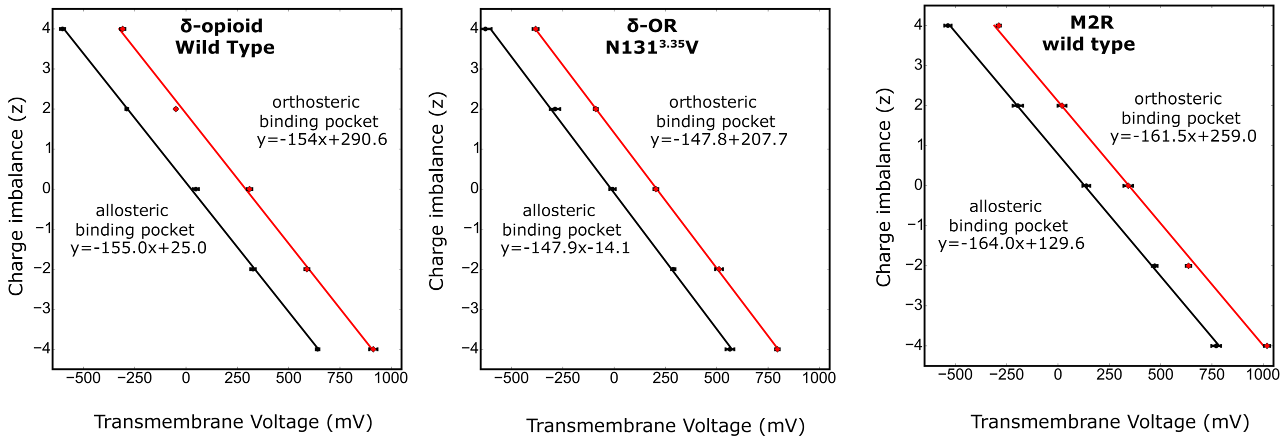

Figure S6 (related to Figure 5 and 6): A linear relationship between transmembrane charge imbalance and V_m exists over a wide voltage range.

The figure shows membrane voltage plotted against charge imbalance, exhibiting a linear relationship. The slope of the curves denotes the capacitance, which is nearly identical in the two states of the ion as can be seen. The voltage displays a right shift (black circles to red squares) due to the movement of the allosteric Na^+ ion from the hydrophilic pocket to the orthosteric ligand binding pocket and further into the extracellular space. From left to right: wt δ-OR, Asn131$^{3.35}$Val δ-OR and wt M2 muscarinic receptor. The examples shown display the effect seen upon movement of a sodium ion from the allosteric site to the transient binding site in the orthosteric pocket. The V_m fluctuations of the system were minimised by the application of position restraints of 3 axes on the protein, and a position restraint of 200kJ/mol nm^2 along the z axis, was applied to the bulk Na^+ to prevent ingress of an additional ion into the orthosteric ligand-binding pocket. In this way, the gating charge arising only from the movement of the allosteric Na^+ ion is recorded. The standard deviation of the variation of V_m falls between 8-30mV using the above protocol for each data point on the graphs, as shown here by the horizontal error bars.

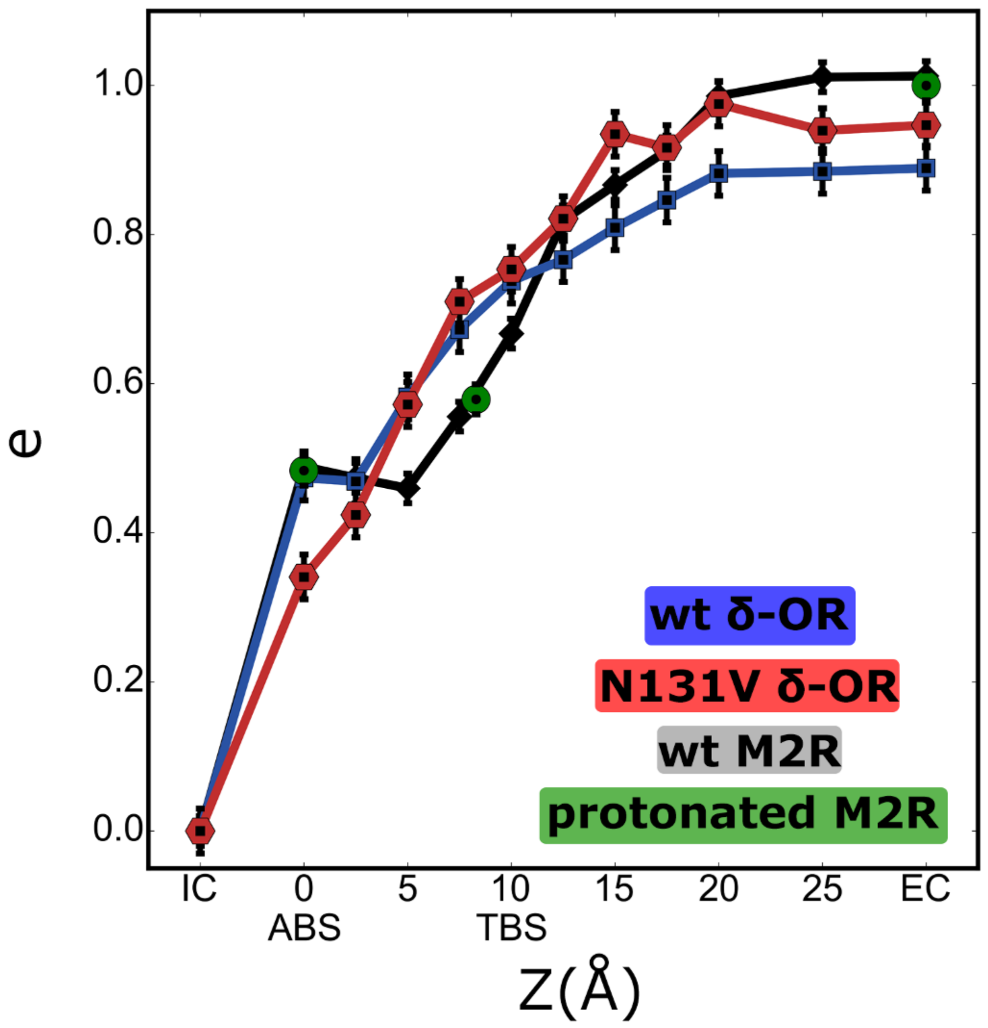

Figure S7 (related to Figure 6): Hypothetical gating charge arising from a complete movement of a Na^+ ion from the intracellular (IC) to extracellular (EC) bulk solution via the orthosteric binding site as a measure of electric field focus.

The maximal gating charges cluster at ~1e with the exception of the wt δ-OR, which shows a slightly lower gating charge resulting from an additional conformational change in N131$^{3.35}$, rotating the amide group. The green circles show the gating charge that would arise from a similar transfer of a proton from the extracellular surface of the M2R (Asp173) to Asp2.50 via Asp3.32. The TM electric field is highly focused between the IC face and the TBS.
